# Controllable population dynamics in Landau-quantized graphene

**DOI:** 10.1038/s41598-017-18176-2

**Published:** 2018-01-24

**Authors:** Chunling Ding, Rong Yu, Xiangying Hao, Duo Zhang

**Affiliations:** 1 School of Science, Wuhan Institute of Technology, Wuhan, 430205 People’s Republic of China; 2 School of Electrical and Electronic Engineering, Wuhan Polytechnic University, Wuhan, 430023 People’s Republic of China

## Abstract

In this paper, we carry out a theoretical investigation on the population dynamics of graphene system under continuous-wave (cw) laser and chirped pulse excitation. Results of our numerical simulations reveal that complete population transfer from an initially occupied ground state to the initially unoccupied excited states can be achieved by choosing appropriate values of the chirp rate, the laser field intensity and frequency, as well as other system parameters. Also, we observe coherent Rabi-like population oscillations between the initial ground state and the excited final state. It is induced by the combined effect of cw and chirped-pulse laser fields. These results will contribute to the understanding of carrier-carrier and carrier-phonon interactions in graphene system, and may find applications in graphene-based high-speed electronic and optoelectronic devices.

## Introduction

The population dynamics of atomic and molecular systems has become a subject of extensive research in recent years, largely motivated by its potential applications in high-performance optical switchings, light emitters, and tunable lasers^1–3^, It has been demonstrated by theoretical and experimental works that atomic systems which exhibit the effect of coherent population oscillations (CPOs) can be utilized as a storage medium for storing light, and this memory is associated with populations and is robust to dephasing effects^4–8^, For more complex molecular structures, the temporal evolution of population in ultracold polar molecules and the measurement of population dynamics in nitrogen-vacancy (NV) centers have been reported^9,10^, In addition, ultrafast excitonic population inversion can be obtained in a hybrid system consisting of quantum dot and metallic nanoparticle, of particular importance is that the system is driven by a nonlinear few-cycle chirped pulse train, and the obtained phenomenon can be used in an ultrafast all-optical switching device^11^.

Quantum coherence and interference effects in atomic and molecular systems have been extensively investigated by means of continuous-wave (cw) laser fields^12–16^, Recent advances in the development of ultrafast optical technology have led to the generation of ultrashort laser pulses. Furthermore, few-cycle laser pulses have been widely used as driving laser fields for investigating atomic coherence and quantum beats^17,18^, electron tunneling and ionization^19,20^, high-order harmonic generation^21–24^, etc. Vala and Kosloff ^25^ presented a coherent mechanism of robust population inversion in atomic and molecular systems by means of a chirped excitation pulse. Compared with atoms and molecules, the graphene is a typical two-dimensional (2D) material composed of carbon atoms in a hexagonal lattice, which has fascinating optical and electrical properties^26–28^, Plasmonic lattice solitons, topological mode switching, nonreciprocal phase shift, Rabi oscillations, dynamic control of coherent pulses, hyper-Raman scattering, as well as optical bistability and multistability have also been investigated in graphene systems^29–37^, The ultrafast dynamics of optically excited carriers in graphene play an important role in the study of quantum many-body physics in 2D materials, such as carrier-carrier interactions and carrier-phonon relaxation processes, since they can be used to explore the underlying physics of high-speed electronic and optoelectronic devices^38–40^.

In the past few years, much effort has been put towards studying carrier dynamics in Landau-quantized graphene under pulsed laser excitation^41–45^, The results showed that the Landau-level dynamics had a strong dependence on the Fermi energy and doping of the graphene sample, as well as the applied magnetic field. All these schemes are excited only by a pulsed laser without considering the continuous laser field. This reminded us of another question: what will the resulting dynamics of graphene system by means of a chirped-pulse-laser field under the simultaneous presence of a cw laser field? In order to address this problem, we put forward a method to study the population dynamics in graphene system under the combined action of cw and chirped-pulse laser fields.

In this work, we derive a system of differential equations which can be used to describe the temporal evolution of Landau-level populations. In order to discuss the properties of population dynamics, two kinds of laser-driven fields are taken into account in our scheme which include continuous and pulsed laser fields. Here, the cw laser field is linearly polarized which can be considered as a superposition of left and right circularly polarized light. On the other hand, for the chirped-pulse laser field, its pulse width enters into the femtosecond time scale. That is to say, we can easily manipulate the population dynamics on the ultrafast processes by utilizing this ultrashort pulsed laser field. By adjusting these tunable parameters, we find that the variation of the chirp rate leads to the change in the final population of four states. The efficiency of population transfer is improved when the dephasing decay rates are neglected. It is also shown that the temporal evolution of Landau-level populations is symmetrical about a point in time when the right and left circularly polarized components of the cw laser fields interact resonantly with the respective transitions. Our investigations may have potential applications in graphene-based high-speed electronic and optoelectronic devices and may bring about substantial impact on related technologies.

## Results

### Theoretical model

**Figure 1:**
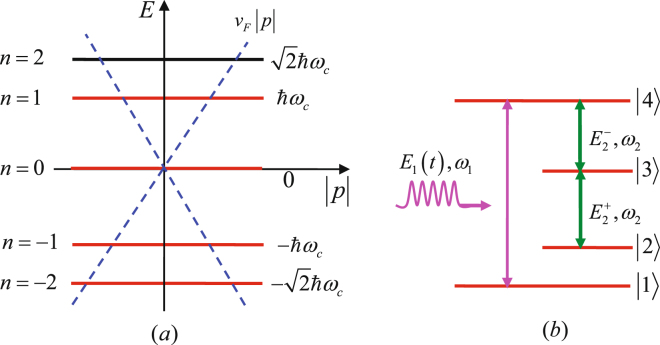
Schematic view of energy bands and optical transitions of graphene system. (**a**) Energy levels near the Dirac point superimposed on the electron dispersion at zero magnetic field (dashed lines), and the energies of Landau levels for a given magnetic field (solid horizontal lines). (**b**) The Landau levels with energy quantum numbers *n* = −2, −1, 0, 1 correspond to the states |1〉, |2〉, |3〉, and |4〉, respectively. A chirped-pulse laser field *E*_1_(*t*) with carrier frequency *ω*_1_ couples the transition |1〉 ↔ |4〉 in Landau-quantized graphene. The right- and left-hand circularly polarized components of a continuous-wave (cw) laser field *E*_2_ with carrier frequency *ω*_2_ drive the transitions |2〉 ↔ |3〉 and |3〉 ↔ |4〉, respectively.

We consider a four-level system identical to the one which has been used to investigate the nonlinear optical response and the generation of entangled photons in graphene^46–48^, as shown in Fig. 1. Similarly, we only take into account the lowest four Landau levels with energy quantum numbers *n* = −2, −1, 0, 1 which can be respectively marked as |1〉, |2〉, |3〉, and |4〉 for convenience of expression. We suppose that the graphene is moderately doped so that the Fermi energy level lies between states |1〉 and |2〉, i.e., the state |1〉 is occupied and the states above are entirely empty without the influence of pumping. Based on the particular selection rules of graphene, Δ|*n*| = ±1, that is, the left-hand circularly polarized light is absorbed when |*n*_*f*_ | = |*n*_*i*_| + 1, conversely, the right-hand circularly polarized light is absorbed when |*n*_*f*_ | = |*n*_*i*_| − 1 with *n*_*f*_ and *n*_*i*_ representing the final and initial energy quantum numbers^49^, we use a chirped-pulse laser field with right-hand circular polarization to couple resonantly the ground state |1〉 to the excited state |4〉. The electric field of the pulsed laser field takes the form

1 $${E}_{1}(t)={E}_{0}\,f(t)\cos \,[{\omega }_{1}t+\phi (t)],$$

here *E*_0_ is the peak amplitude of the electric field envelope, the field envelope can be written as $f(t)=\exp$ $[-{(t-2\tau )}^{2}/{\tau }^{2}]$ with *τ* being the pulse duration defined by the full width at half maximum (FWHM) of the field *E*_1_(*t*), and *ω*_1_ denotes the carrier frequency of chirped-pulse laser field. Besides, $\phi (t)=-\eta \,\cosh \,[(t-2\tau )/{\tau }_{c}]$ is the time-varying carrier-envelope phase (CEP), which represents the time-dependent offset between the peak of laser pulse and the peak position of the Gaussian envelope. The shape of the chirped pulse is dependent on the frequency-sweep range *η* and the steepness of the chirp *τ*_*c*_. Following the rapid progress in the field of comb laser technology, it is possible to achieve such a time-changing CEP in the near future^50^. Simultaneously, the right- and left-hand circularly polarized components of a linearly polarized cw laser field with carrier frequency *ω*_2_ are used to couple the transitions |2〉 → |3〉 and |3〉 → |4〉, respectively. The electric field of the cw laser field can be denoted by ${\overrightarrow{E}}_{2}=({\hat{e}}_{2}^{+}{E}_{2}^{+}+{\hat{e}}_{2}^{-}{E}_{2}^{-})\exp (-i{\omega }_{2}t+i{\overrightarrow{k}}_{2}\cdot \overrightarrow{r})+c\mathrm{.}\,c\mathrm{.}$, here ${\hat{e}}_{2}^{+}=[\hat{x}+i\hat{y}]/\sqrt{2}$ and ${\hat{e}}_{2}^{-}=[\hat{x}-i\hat{y}]/\sqrt{2}$ are, respectively, the unit vectors of the right- and left-hand circularly polarized basis.

### Temporal evolution of Landau-level populations

Our goal in this work is to discuss the population dynamics of graphene system by varying the system parameters, such as the chirp rate, the dephasing decay rate, the laser field intensity and frequency. Before proceeding further, we consider a suitable parameter value for *γ*_4_ = 0.03 fs^−1^ based on the numerical estimate in ref ^51^. Also, we take the same value for ${\omega }_{c}\simeq 0.1$ fs^−1^ at the magnetic field strength of *B* = 3 T as that shown in refs^46–48^, For simplicity but without loss of generality, we assume throughout this paper that ${{\rm{\Omega }}}_{2}^{+}={{\rm{\Omega }}}_{2}^{-}={{\rm{\Omega }}}_{2}$.

**Figure 2:**
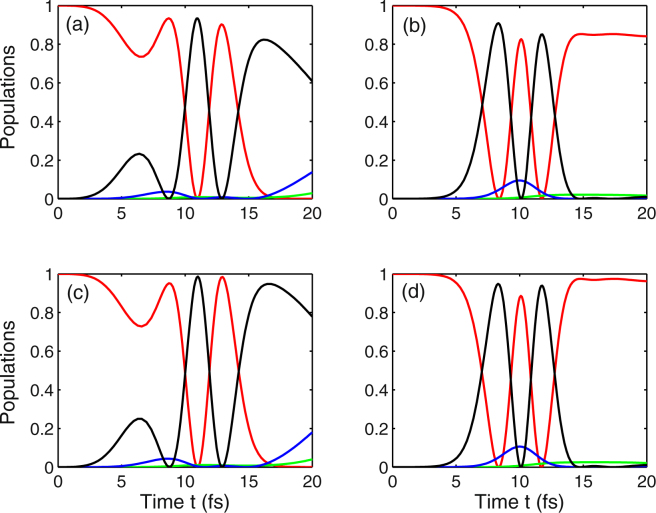
Temporal evolution of populations *ρ*_11_ (red curve), *ρ*_22_ (green curve), *ρ*_33_ (blue curve), and *ρ*_44_ (black curve). For the numerical simulations, we choose the following parameter values: (**a**) *η* = 0, (*γ*_2_,*γ*_3_,*γ*_4_) = (0.003,0.03,0.03) fs^−1^; (**b**) *η* = −0.5 rad, (*γ*_2_,*γ*_3_,*γ*_4_) = (0.003,0.03,0.03) fs^−1^; (**c**) *η* = 0, (*γ*_2_,*γ*_3_,*γ*_4_) = (0,0,0); (**d**) *η* = −0.5 rad, (*γ*_2_,*γ*_3_,*γ*_4_) = (0,0,0). Other parameters are Ω_1_ = 1 fs^−1^, Ω_2_ = 0.1 fs^−1^, *B* = 3 T, *ω*_*c*_ = 0.1 fs^−1^, *ω*_1_ = 0.24 fs^−1^, *ω*_2_ = 0.1 fs^−1^, *τ* = 5 fs, and *τ*_*c*_ = 4.84 fs.

Firstly, we discuss the role of the chirp rate and the dephasing decay rate in achieving population inversion between the Landau levels. Specifically we explore the following four cases: (a) chirp-free with considering decay rate, (b) chirped pulse and considering decay rate, (c) chirp-free pulse and neglecting decay rate, as well as (d) chirped-pulse laser and without the decay rate. Taking into account the chirp-free pulse and dephasing decay rate, such that *η* = 0 and (*γ*_2_,*γ*_3_,*γ*_4_) = (0.003,0.03,0.03) fs^−1^, we numerically solve Eq. 3(a–j) of the Methods for the investigation of population dynamics in Landau-quantized graphene [see Fig. 2(a)]. This figure clearly shows that, for a realistic set of parameters, population inversion near the Dirac point can be achieved within ten femtoseconds after optical excitation. And we prove, the same condition holds under femtosecond chirped pulse excitation, that the ultrafast population inversion between the initially occupied ground state |1〉 (red curve) and the initially unoccupied excited state |4〉 (black curve) takes place before and after *t* = 2*τ* as depicted in Fig. 2(b). When the dephasing decay rate is neglected, the similar behavior of population dynamics can be observed in Fig. 2(c,d), except that the efficiency of population transfer is improved and reaches almost unity. Comparison between the two cases: chirp-free [i.e., *η* = 0 in Fig. 2(a,c)] and chirped-pulse [i.e., *η* = −0.5 rad in Fig. 2(b,d)], we find that the final population (i.e., *t* = 4*τ*) is different. More specifically, when *η* = 0, the population is mainly distributed in the state |4〉 (black curve), and less occupation numbers are distributed in the state |3〉 (blue curve) as shown in Fig. 2(a,c). Whereas, in the case of *η* = −0.5 rad, the final population of the state |3〉 is completely suppressed, and there is a little distribution in this state at time *t* = 2*τ* as displayed in Fig. 2(b,d). It is worth noticing that the temporal evolution of populations in states |1〉, |3〉, and |4〉 exhibits a symmetrical distribution with respect to the point *t* = 2*τ* under pulsed laser excitation and ignoring the dephasing decay rate [see Fig. 2(d)].

**Figure 3:**
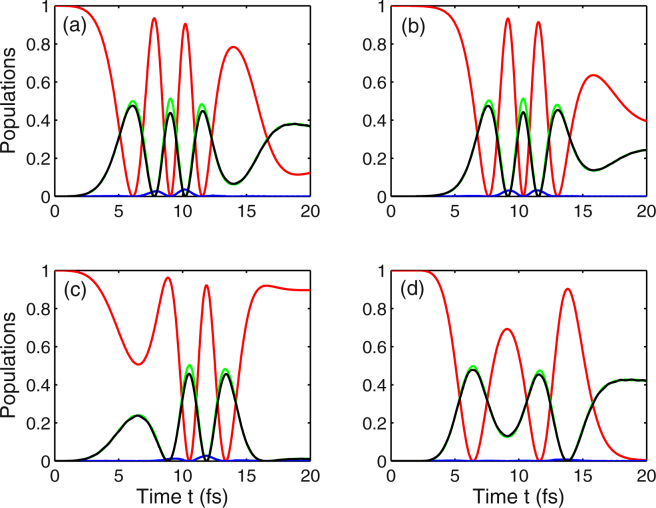
Temporal evolution of populations *ρ*_11_ (red curve), *ρ*_22_ (green curve), *ρ*_33_ (blue curve), and *ρ*_44_ (black curve) for the four different values of the chirp rate. (**a**) *η* = ^−1^ rad; (**b**) *η* = −0.5 rad; (**c**) *η* = 0; (**d**) *η* = 0.6 rad. Other parameters are Ω_1_ = 2 fs^−1^, Ω_2_ = 5 fs^−1^, *B* = 3 T, *ω*_*c*_ = 0.1 fs^−1^, *ω*_1_ = 0.24 fs^−1^, *ω*_2_ = 0.1 fs^−1^, *γ*_2_ = 0.003 fs^−1^, *γ*_3_ = *γ*_4_ = 0.03 fs^−1^, *τ* = 5 fs, and *τ*_*c*_ = 4.84 fs.

Next, we investigate the effect of chirp parameter on the behavior of population dynamics in Landau-quantized graphene. Under the influence of a negative chirped laser field, the phenomenon that ultrafast population transfer from a single initial state |1〉 (red curve) to a superposition of two excited states |2〉 (green curve) and |4〉 (black curve) occurs three times within a period of ten femtoseconds, as can be seen from Fig. 3(a). Comparing the case of *η* = −0.5 rad [see Fig. 3(b)] with *η* = −1 rad [see Fig. 3(a)], we find that the spectrum of population inversion is shifted to the right, which means that the achievement of population inversion is delayed. Moreover, the final population differs from each other in these two cases. It should be noted that the spectrum of population inversion is progressively shifted towards the right and the complete population inversion appears twice within the same time under the condition of a chirp-free pulsed field [i.e., *η* = 0 in Fig. 3(c)]. Besides, we find a similar behavior for the population dynamics illustrated in Fig. 3(d), the difference is that the complete population inversion in the presence of a positive chirped field appears ahead of that in the presence of chirp-free pulsed field. And the final population in Fig. 3(d) is different from that shown in Fig. 3(c) because of the existence of the chirped laser field. However, our numerical results indicate that, for the non-chirped and negative chirped pulses, there is a little of population distributed on the state |3〉, whereas the population of state |3〉 is completely inhibited under a positive chirped laser irradiation, as shown by the blue curves in Fig. 3.

**Figure 4:**
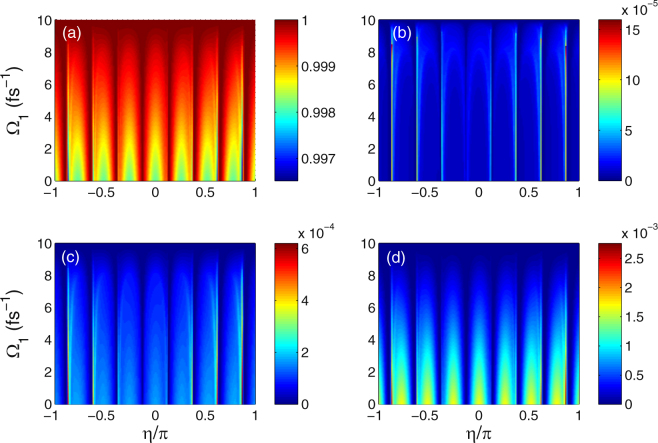
Color-coded plot of the populations as functions of the Rabi frequency Ω_1_ and sweeping parameter *η*/*π* at time *t* = 4*τ* = 20 fs. (**a**) *ρ*_11_(4*τ*), (**b**) *ρ*_22_(4*τ*), (**c**) *ρ*_33_(4*τ*), and (**d**) *ρ*_44_(4*τ*). Other parameters are Ω_2_ = 3 fs^−1^, *B* = 3 T, *ω*_*c*_ = 0.1 fs^−1^, *ω*_1_ = 0.24 fs^−1^, *ω*_2_ = 0.1 fs^−1^, *γ*_2_ = 0.003 fs^−1^, *γ*_3_ = *γ*_4_ = 0.03 fs^−1^, *τ* = 5 fs, and *τ*_*c*_ = 4.84 fs.

Figure 4 shows the color-coded plots of the population as functions of the Rabi frequency Ω_1_ and sweeping parameter *η*/*π* at time *t* = 4*τ*. The striking feature in Fig. 4 is that the distribution of final population changes periodically with the variation of sweeping parameter *η*, and which is symmetrical with respect to *η* = 0. The spectral distributions of *ρ*_11_(4*τ*) and *ρ*_44_(4*τ*) are similar, but color does the opposite, as shown in Fig. 4(a) and (d). Note that the spectrum profiles in graphs 4(b) and (c) are almost identical except for a slight difference in color. The underlying physical mechanism for the creation of population inversion between the initially occupied and empty levels is that there exists quantum interference among multiple transition pathways induced by the combined effect of cw and chirped-pulse laser fields. Moreover, the distribution of final population varies periodically because of the application of periodically transmitting pulsed field.

### Coherent manipulation of population dynamics

**Figure 5:**
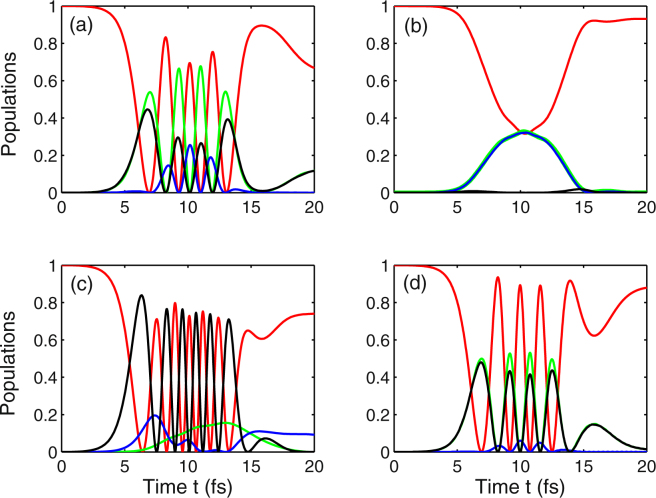
Temporal evolution of populations *ρ*_11_ (red curve), *ρ*_22_ (green curve), *ρ*_33_ (blue curve), and *ρ*_44_ (black curve) for different values of laser field intensity and frequency. (**a**) Ω_2_ = 3 fs^−1^, *ω*_2_ = 0.1 fs^−1^; (**b**) Ω_2_ = 3 fs^−1^, *ω*_2_ = 3.1 fs^−1^; (**c**) Ω_2_ = 0.2 fs^−1^, *ω*_2_ = 0.1 fs^−1^; (**d**) Ω_2_ = 6 fs^−1^, *ω*_2_ = 0.1 fs^−1^. Other parameters are Ω_1_ = 3 fs^−1^, *B* = 3 T, *ω*_*c*_ = 0.1 fs^−1^, *ω*_1_ = 0.24 fs^−1^, *γ*_2_ = 0.003 fs^−1^, *γ*_3_ = *γ*_4_ = 0.03 fs^−1^, *η* = −0.5 rad, *τ* = 5 fs, and *τ*_*c*_ = 4.84 fs.

Now we turn our attention to the analysis of coherent manipulation of population dynamics by properly adjusting intensity and frequency of the cw laser field. When the cw laser field is tuned into resonance with the corresponding transition and the field intensity is taken the same value as the pulsed laser field, we can see from Fig. 5(a) that the evolution spectrum of the state population can be approximated to be symmetrical about *t* = 2*τ*, and part of populations are distributed in the states |2〉 (green curve) and |4〉 (black curve) at the end of the pulse. In other words, after five femtoseconds, the population transfer from the initially occupied state |1〉 (red curve) to the initial empty state |4〉 (black curve), and simultaneously, most of populations in the excited state |4〉 (black curve) decay into the lower-lying excited state |3〉 (blue curve), and then quickly decay to the state |2〉 (green curve), as a result, these is a little population distributed in the state |3〉, as illustrated in Fig. 5(a). When the cw laser field is far away from the resonant Landau-level transition and its intensity remains unchanged, only part of population transfers from the ground state |1〉 (red curve) to the upper states |3〉 (blue curve) and |2〉 (green curve) at around *t* = 2*τ*, which is presented in Fig. 5(b), and the population in the excited state |4〉 is completely relaxed to the nearest-neighbor level as compared with the resonant condition sketched in Fig. 5(a). Further, we discuss two other options for the intensity of cw laser field under resonant excitation shown in Fig. 5(c) and (d). We find that, when the intensity of cw laser field is much smaller than the pulsed field, 80 percent of population exhibits Rabi-like oscillations between the initial state |1〉 (red curve) and the excited final state |4〉 (black curve), and only 20 percent of population oscillations between other two states |3〉 (blue curve) and |2〉 (green curve) as displayed in Fig. 5(c). On the contrary, when the intensity of cw laser field is greater than the pulsed field, we obtain a similar result to that shown in Fig. 5(a), the difference is that the periodic oscillation of population is no longer an envelope shape, and the population oscillations without any attenuation can be observed in Fig. 5(d). The appearance of Rabi-like oscillations between the initial and final states is due to the fact that the recombination occurs between the intraband and interband transitions which is caused by the carrier-carrier Coulomb interaction and carrier-phonon interaction in the vicinity of the Dirac point in graphene.

**Figure 6:**
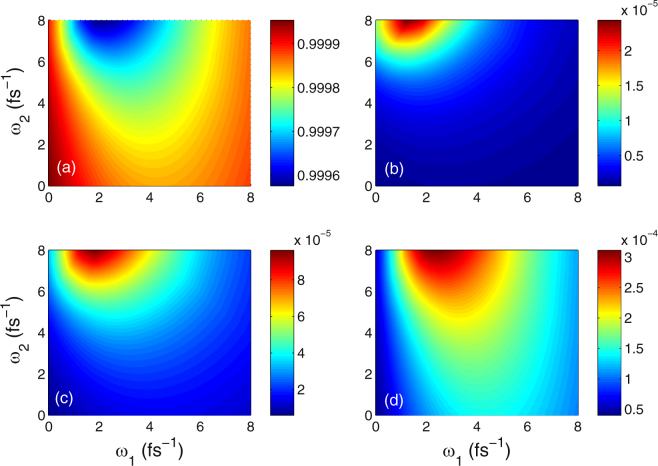
Color-coded plot of the populations as functions of the laser frequencies *ω*_1_ and *ω*_2_ at time *t* = 4*τ* = 20 fs. (**a**) *ρ*_11_(4*τ*), (**b**) *ρ*_22_(4*τ*), (**c**) *ρ*_33_(4*τ*), and (**d**) *ρ*_44_(4*τ*). Other parameters are Ω_1_ = Ω_2_ = 3 fs^−1^, *B* = 3 T, *ω*_*c*_ = 0.1 fs^−1^, *γ*_2_ = 0.003 fs^−1^, *γ*_3_ = *γ*_4_ = 0.03 fs^−1^, *η* = −0.5 rad, *τ* = 5 fs, and *τ*_*c*_ = 4.84 fs.

In Fig. 6 we show the color-coded plot of the variation of the final population in Landau-quantized graphene. The distribution of final population is marked by different colors. It should be noted that the patterns of Fig. 6(a) and Fig. 6(b–d) are almost identical, but with the opposite coloring. This indicates that part of population transfers from the initially occupied ground state |1〉 to the initially unoccupied upper states |*j*〉 (*j* = 2, 3, 4) under the combined action of cw and chirped-pulse laser fields. Yet, slight difference remains for the color of Fig. 6(b) and (c). That is to say, the difference between them originates from the variation of laser frequency.

Before ending this section, let us briefly discuss the differences between our current and previous work^52^. (i) In our previous work, we used two opposite circularly polarized components of a few-cycle laser pulse with linear polarization to couple two different intraband Landau-level transitions. However, in the present paper, the chirped-pulse laser field with right-hand circular polarization is applied to couple the interband Landau-level transition, the right- and left-hand circularly polarized components of a linearly polarized cw laser field are employed to couple the intraband transitions between Landau levels. (ii) Our previous work did not analyze the influence of the chirp rate on population dynamics. In this work, we discuss in detail the effects of the chirp rate on the behavior of population dynamics in Landau-quantized graphene. (iii) In addition to the similar results such as Rabi-like oscillations and interband population inversion, we also found that the appearance of complete population inversion can be controlled by the chirp parameter in our present work.

## Discussion

We have theoretically investigated the generation of complete population inversion in Landau-quantized graphene under the combined action of cw and chirped-pulse laser fields. By using numerical simulations, the influence of two types of laser fields parameters including intensity, frequency, and the chirp rate on the population dynamics of the system has been studied. It has been demonstrated that the consideration of sweeping parameter, in addition to the effect of cw laser field interaction, considerably changes the population distribution. We have found that the efficiency of attainable population transfer between the initially occupied ground state and the initially unoccupied excited state is improved in the absence of dephasing rate. However, it has been shown that the efficient population inversion can also be achieved even though the decay rate is taken into account. The obtained results show that some specific behaviors of the state population can be observed, such as the Rabi-like oscillations, periodicity and symmetry of the population evolution. These phenomena can be attributed to carrier-carrier and carrier-phonon interactions induced by the cw and chirped-pulse laser fields. Also, it should be mentioned that our findings may have potential applications in graphene-based high-speed electronic and optoelectronic devices, and may contribute to the design and development of related technologies.

## Methods

### Derivation of the system of differential equations

Substituting the interaction Hamiltonian that has been constructed in refs^46–48^ into the Liouville equation.

2 $$\dot{\rho }=-\frac{i}{\hslash }[{H}_{int},\rho ]-\frac{1}{2}\{{\rm{\Gamma }},\rho \},$$

here {Γ, *ρ*} = Γ*ρ* + *ρ*Γ. It should be noted that the relaxation matrix Γ in Eq. 2 denotes the decay rate, which can be given by 〈*n*|Γ|*m*〉 = *γ*_*n*_*δ*_*mn*_ with *γ*_*n*_ being the dephasing decay rate. Accordingly, the system of differential equations for describing the temporal evolution of population in graphene system can be obtained as follows

3a $${\dot{\rho }}_{11}=i{{\rm{\Omega }}}_{1}\,f(t)\cos \,[{\omega }_{1}t+\phi (t)]({\rho }_{41}-{\rho }_{14}),$$

3b $${\dot{\rho }}_{22}=i{{\rm{\Omega }}}_{2}^{+}({\rho }_{32}-{\rho }_{23})-{\gamma }_{2}{\rho }_{22},$$

3c $${\dot{\rho }}_{33}=i{{\rm{\Omega }}}_{2}^{+}({\rho }_{23}-{\rho }_{32})+i{{\rm{\Omega }}}_{2}^{-}({\rho }_{43}-{\rho }_{34})-{\gamma }_{3}{\rho }_{33},$$

3d $${\dot{\rho }}_{44}=i{{\rm{\Omega }}}_{1}\,f(t)\cos \,[{\omega }_{1}t+\phi (t)]({\rho }_{14}-{\rho }_{41})+i{{\rm{\Omega }}}_{2}^{-}({\rho }_{34}-{\rho }_{43})-{\gamma }_{4}{\rho }_{44},$$

3e $${\dot{\rho }}_{21}=-i({\omega }_{32}-{\omega }_{2}-i\frac{{\gamma }_{2}}{2}){\rho }_{21}+i{{\rm{\Omega }}}_{2}^{+}{\rho }_{31}-i{{\rm{\Omega }}}_{1}\,f(t)\cos \,[{\omega }_{1}t+\phi (t)]{\rho }_{24},$$

3f $${\dot{\rho }}_{31}=-i({\omega }_{43}-{\omega }_{2}-i\frac{{\gamma }_{3}}{2}){\rho }_{31}+i{{\rm{\Omega }}}_{2}^{+}{\rho }_{21}+i{{\rm{\Omega }}}_{2}^{-}{\rho }_{41}-i{{\rm{\Omega }}}_{1}\,f(t)\cos \,[{\omega }_{1}t+\phi (t)]{\rho }_{34},$$

3g $${\dot{\rho }}_{41}=-i({\omega }_{41}-{\omega }_{1}-i\frac{{\gamma }_{4}}{2}){\rho }_{41}+i{{\rm{\Omega }}}_{1}f(t)\cos \,[{\omega }_{1}t+\phi (t)]({\rho }_{11}-{\rho }_{44})+i{{\rm{\Omega }}}_{2}^{-}{\rho }_{31},$$

3h $${\dot{\rho }}_{32}=i{{\rm{\Omega }}}_{2}^{+}({\rho }_{22}-{\rho }_{33})+i{{\rm{\Omega }}}_{2}^{-}{\rho }_{42}-\frac{{\gamma }_{3}+{\gamma }_{2}}{2}{\rho }_{32},$$

3i $$\begin{array}{c}{\dot{\rho }}_{42}=-i({\omega }_{41}-{\omega }_{32}-{\omega }_{1}+{\omega }_{2}-i\frac{{\gamma }_{4}+{\gamma }_{2}}{2}){\rho }_{42}+i{{\rm{\Omega }}}_{1}\,f(t)\cos \,[{\omega }_{1}t+\phi (t)]{\rho }_{12}\\ \quad \quad \,+i{{\rm{\Omega }}}_{2}^{-}{\rho }_{32}-i{{\rm{\Omega }}}_{2}^{+}{\rho }_{43},\end{array}$$

3j $$\begin{array}{c}{\dot{\rho }}_{43}=-i({\omega }_{41}-{\omega }_{43}-{\omega }_{1}+{\omega }_{2}-i\frac{{\gamma }_{3}+{\gamma }_{4}}{2}){\rho }_{43}+i{{\rm{\Omega }}}_{1}\,f(t)\cos \,[{\omega }_{1}t+\phi (t)]{\rho }_{13}\\ \quad \quad \,+i{{\rm{\Omega }}}_{2}^{-}({\rho }_{33}-{\rho }_{44})-i{{\rm{\Omega }}}_{2}^{+}{\rho }_{42},\end{array}$$

where, ${{\rm{\Omega }}}_{1}=({\overrightarrow{\mu }}_{41}\cdot {\overrightarrow{E}}_{1}\mathrm{)/(2}\hslash )$, ${{\rm{\Omega }}}_{2}^{+}=({\overrightarrow{\mu }}_{32}\cdot {\overrightarrow{E}}_{2}^{+}\mathrm{)/(2}\hslash ),$ and ${{\rm{\Omega }}}_{2}^{-}=({\overrightarrow{\mu }}_{43}\cdot {\overrightarrow{E}}_{2}^{-}\mathrm{)/(2}\hslash )$ represent one-half Rabi frequencies for the respective transitions, here ${\overrightarrow{{\mu }}}_{mn}=|\langle m|\overrightarrow{{\mu }}|n\rangle |=e\cdot \langle m|\overrightarrow{r}|n\rangle \,=\,\frac{i\hslash e}{{\varepsilon }_{n}-{\varepsilon }_{m}}\langle m|{v}_{F}\overrightarrow{\sigma }|n\rangle$ indicates the corresponding dipole matrix element. ${\varepsilon }_{n}={\rm{sgn}}(n)\hslash {\omega }_{{\rm{c}}}\sqrt{|n|}$ is the energy of the Landau levels for electrons or holes close to the Dirac point^46–48^, with *n* = 0, ±1, ±2,…, ${{w}}_{c}=\sqrt{2{v}_{{F}}}/{l}_{c},$ and ${l}_{c}=\sqrt{\hslash /eB}$ is the magnetic length. Additionally, the value of the band parameter (Fermi velocity) can be taken as *v*_*F*_ ≈ 10^6^*m*/*s*. $\overrightarrow{\sigma }=({\hat{\sigma }}_{x},{\hat{\sigma }}_{y})$ is the Pauli matrix vector.
